# Lipopolysaccharide induces neuroglia activation and NF-κB activation in cerebral cortex of adult mice

**DOI:** 10.1186/s42826-019-0018-9

**Published:** 2019-10-16

**Authors:** Ju-Bin Kang, Dong-Ju Park, Murad-Ali Shah, Myeong-Ok Kim, Phil-Ok Koh

**Affiliations:** 1 Department of Anatomy, College of Veterinary Medicine, Research Institute of Life Science, 501 Jinju-daero, Jinju, 52828 South Korea; 2 Division of Life Science and Applied Life Science, College of Natural Sciences, Gyeongsang National University, 501 Jinju-daero, Jinju, 52828 South Korea

**Keywords:** Lipopolysaccharide, Neuroinflammation, Nuclear factor kappa B, Oxidative stress, Reactive oxygen species

## Abstract

Lipopolysaccharide (LPS) acts as an endotoxin, releases inflammatory cytokines, and promotes an inflammatory response in various tissues. This study investigated whether LPS modulates neuroglia activation and nuclear factor kappa B (NF-κB)-mediated inflammatory factors in the cerebral cortex. Adult male mice were divided into control animals and LPS-treated animals. The mice received LPS (250 μg/kg) or vehicle via an intraperitoneal injection for 5 days. We confirmed a reduction of body weight in LPS-treated animals and observed severe histopathological changes in the cerebral cortex. Moreover, we elucidated increases of reactive oxygen species and oxidative stress levels in LPS-treated animals. LPS administration led to increases of ionized calcium-binding adaptor molecule-1 (Iba-1) and glial fibrillary acidic protein (GFAP) expression. Iba-1 and GFAP are well accepted as markers of activated microglia and astrocytes, respectively. Moreover, LPS exposure induced increases of NF-κB and pro-inflammatory factors, such as interleukin-1β (IL-1β) and tumor necrosis factor-α (TNF-α). Increases of these inflammatory mediators by LPS exposure indicate that LPS leads to inflammatory responses and tissue damage. These results demonstrated that LPS activates neuroglial cells and increases NF-κB-mediated inflammatory factors in the cerebral cortex. Thus, these findings suggest that LPS induces neurotoxicity by increasing oxidative stress and activating neuroglia and inflammatory factors in the cerebral cortex.

## Introduction

Lipopolysaccharide (LPS) is known as a lipoglycan and endotoxin that is found in the outer membrane of gram-negative bacteria. LPS administration induces cognitive impairment and behavioral depression [1, 2]. It also exacerbates the extent of brain damage after an experimental stroke [3]. Moreover, it induces neuroinflammation and neurodegeneration in mice by stimulating pro-inflammatory cytokines [4, 5]. LPS increases oxidative stress, releases inflammatory cytokines, and induces inflammatory response [6, 7]. Inflammation is a complex biological response against harmful stimuli such as pathogens. It performs a critical role in removing the initial cause of cell injury, clearing out damaged cells, and initiating tissue repair. Ionized calcium-binding adapter molecule 1 (Iba-1) is a calcium/actin-binding protein that is specifically expressed in macrophages and microglia [8]. Iba-1 is induced by cytokines and interferons and is involved in the inflammatory response [9]. Moreover, glial fibrillary acidic protein (GFAP) is an intermediate filament protein that is mainly expressed in astrocytes [10]. It is involved in many important central nervous system processes, including cell motility and blood-brain barrier formation [11, 12]. Thus, Iba-1 and GFAP are accepted as markers of microglia and astrocytes.

Nuclear factor kappa B (NF-κB) is a protein complex that controls cytokine production and cell survival [13]. NF-κB regulates cellular processes including cell proliferation, apoptosis, and inflammatory response [14–16]. It is involved in cellular responses to stimuli, such as free radicals, cytokines, and bacterial or viral antigens [17–19]. Moreover, NF-κB has pro-inflammatory properties. It induces gene expression of pro-inflammatory cytokines, such as interleukin 1 β (IL-1β) and tumor necrosis factor- α (TNF-α). IL-1β is a member of the interleukin 1 family of cytokines [20]. TNF-α is a member of the tumor necrosis factor superfamily, which is a cytokine involved in systemic inflammation [21]. These cytokines are produced by activated macrophages and are involved in various cellular activities, including cell differentiation, proliferation, and apoptosis [22–25]. Moreover, these cytokines are well known as important mediators of the inflammatory response. We propose that LPS can lead to an inflammatory response through regulation of NF-κB, IL-1β, and TNF-α in the cerebral cortex. Thus, we investigated whether LPS modulates activations of neuroglial cells and NF-κB mediated inflammatory factors in the cerebral cortex of adult mice.

## Materials and methods

### Experimental animal and drug treatment

Male BALB/c mice (6 weeks, 33–35 g, *n* = 30) were supplied from Samtako Co. (Animal Breeding Centre, Osan, Korea). All animal experiments were performed in accordance with guidelines of the Institutional Animal Care and Use Committee of Gyeongsang National University. Mice were housed in controlled temperature (25 ± 2 °C) with a 12 h light/12 h dark cycle and provided with free access to feed and water. Animals were randomized into control and LPS-treated groups. LPS (250 μg/kg, Sigma Aldrich, St. Louis, MO, USA) was dissolved in normal saline and intraperitoneally injected for 5 days [26, 27]. Control animals were administered with the same volume of normal saline. Body weights of mice were measured on every morning and mice were sacrificed at 24 h after last injection. Brain tissues were separated from skull and fixed in 4% formaldehyde for histological analysis. Moreover, cerebral cortices were quickly isolated from brain and stored at − 70 °C for reactive oxygen species (ROS), lipid peroxidation, and Western blot analyses.

### Reactive oxygen species (ROS) assay

Cerebral cortices were homogenized in lysis buffer [1% Triton X-100, 1 mM EDTA in phosphate buffer saline (PBS, pH 7.4)] and centrifuged at 15,000 g for 20 min. Supernatants were isolated and protein concentration was measured with bicinchoninic acid (BCA) protein analysis kit (Pierce, Rockford, IL, USA). Samples were diluted with Locke’s buffer [154 mM NaCl, 5.6 mM KCl, 3.6 mM NaHCO_3_, 2.0 mM CaCl_2_, 10 mM D-glucose, and 5 mM 4-(2-hydroxyethyl)-1-piperazineethanesulfonic acid] at 5 mg/ml. 2′,7′-dichlorodihydrofluorescein diacetate (DCFH-DA, Sigma Aldrich), which can be oxidized into fluorescent dichlorofluorescein (DCF), was used to detect the formation of ROS. Diluted samples were blended into 5 mM DCFH-DA and incubated for 15 min at room temperature. ROS generations were observed at excitation wavelength 484 nm and emission wavelength 530 nm using a spectrofluorimeter. ROS analysis was expressed as DCF pmol/mg of protein. ROS value of control group was set to 1.

### Lipid peroxidation (LPO) assay

LPO assay was measured with malondialdehyde (MDA) that is production of lipid peroxidation. It was followed by manufacturer’s instruction (BioVision Inc., Milpitas, CA, USA). Cerebral cortices were homogenized with MDA lysis buffer and butylated hydroxytoluene on ice. Samples were centrifuged in 13,000 g for 10 min to remove insoluble material and supernatants were collected. Supernatants were incubated with thiobarbituric acid at 95 °C for 60 min and cooled in ice for 10 min. Absorbance of supernatants were read at 532 nm and MDA amount was expressed as nmol/mg of protein. MDA value of control group was set to 1.

### Hematoxylin and eosin staining

Fixed brain tissues were washed with tap water for overnight, dehydrated by gradient of ethyl alcohol from 70 to 100%, and cleaned with xylene. Tissues were embedded with paraffin using embedding center (Leica, Wetzlar, Germany), sectioned into 4 μm coronal section on a rotary microtome (Leica), placed on slide glass, and dried on a slide warmer (Thermo Fisher Scientific, Waltham, MA, USA). Sections were depaffinized with xylene, rehydrated by gradient of ethyl alcohol from 100 to 70%, and stained with Harris’ hematoxylin solution (Sigma-Aldrich) and eosin Y solution (Sigma Aldrich). Stained sections were dehydrated by gradient of ethyl alcohol from 70 to 100%, cleaned with xylene, and mounted with permount mounting solution (Thermo Fisher Scientific). They were observed and photographed with Olympus microscope (Olympus, Tokyo, Japan).

### Western blot analysis

Protein samples of cerebral cortices were extracted with lysis buffer [1% Triton X-100, 1 mM EDTA in 1 × PBS (pH 7.4)] containing 200 μM phenylmethylsulfonyl fluoride and centrifuged at 15,000 g for 20 min. Supernatants were collected and protein concentrations were measured using a BCA protein analysis kit (Pierce). Protein samples (30 μg) were denatured for 3 min at 100 °C, incubated in ice for 1 min, and electrophoresed in 10% sodium dodecyl sulfate poly-acrylamide gels. Loaded samples were transferred to polyvinylidenedifluoride membranes (Millipore, Billerica, MA, USA). Membranes were blocked with 5% skim milk solution diluted in Tris-buffered saline containing 0.1% Tween-20 (TBST) for 1 h and washed with TBST. After washing, membranes were incubated for overnight at 4 °C with following primary antibody: anti-Iba-1, anti-GFAP, anti-NF-κB, anti-TNF-α, anti-IL-1β, and anti-β-actin (anti-Iba-1, anti-GFAP, anti-NF-κB, anti-TNF-α, anti-IL-1β, and anti-β-actin primary antibody, diluted 1:1000, Santa Cruz Biotechnology, Dallas, TX, USA). Membranes were washed with TBST and incubated with horseradish peroxidase-conjugated anti-mouse IgG or anti-rabbit IgG (1:5000, Cell Signaling Technology) for 2 h at room temperature. After washing with TBST, membranes were reacted with enhanced chemiluminescence detection reagents (GE Healthcare, Little Chalfont, Buckinghamshire, UK) for detecting immunoreactive protein bands and exposed to Fuji medical X-ray film (Fuji Film, Tokyo, Japan).

### Immunofluorescence staining

Paraffin sections were deparaffinied with xylene and rehydrated by gradient of ethyl alcohol from 100 to 70%. Sections were washed with PBS and reacted with normal goat serum for 1 h for blocking non-specific reaction. Sections were incubated in a wet chamber for overnight at 4 °C with following primary antibodies: anti-Iba-1, anti-GFAP, anti-NF-κB, anti-TNF-α, and anti-IL-1β (anti-Iba-1, anti-GFAP, anti-NF-κB, anti-TNF-α, and anti-IL-1β primary antibody, diluted 1:100, Santa Cruz Biotechnology). Sections were washed with PBS and incubated with fluorescein isothiocyanate (FITC)-conjugated anti-mouse IgG or anti-rabbit IgG (1:200, Santa Cruz Biotechnology) for 2 h at room temperature. Sections were reacted with 4′,6-diamidino-2-phenylindole (DAPI, Sigma Aldrich) for 10 min and mounted with Ultra-Cruz mounting medium (Santa Cruz Biotechnology). Fluorescent signals were observed and imaged with a confocal microscope (FV-1000, Olympus, Tokyo, Japan). Integrated intensities of positive signals were analyzed by Image-Pro Plus image software (Media Cybernetics, Rockville, MD, USA). Intensity values were expressed as a ratio of LPS-treated group intensity to control group intensity. Intensity value of control group was set to 1.

### Statistical analysis

Data are presented as mean ± standard error of mean (S.E.M.). Results of group were compared by Student’s *t*-test. A value of *p* < 0.05 was statistically considered significant.

## Results

**Fig. 1:**
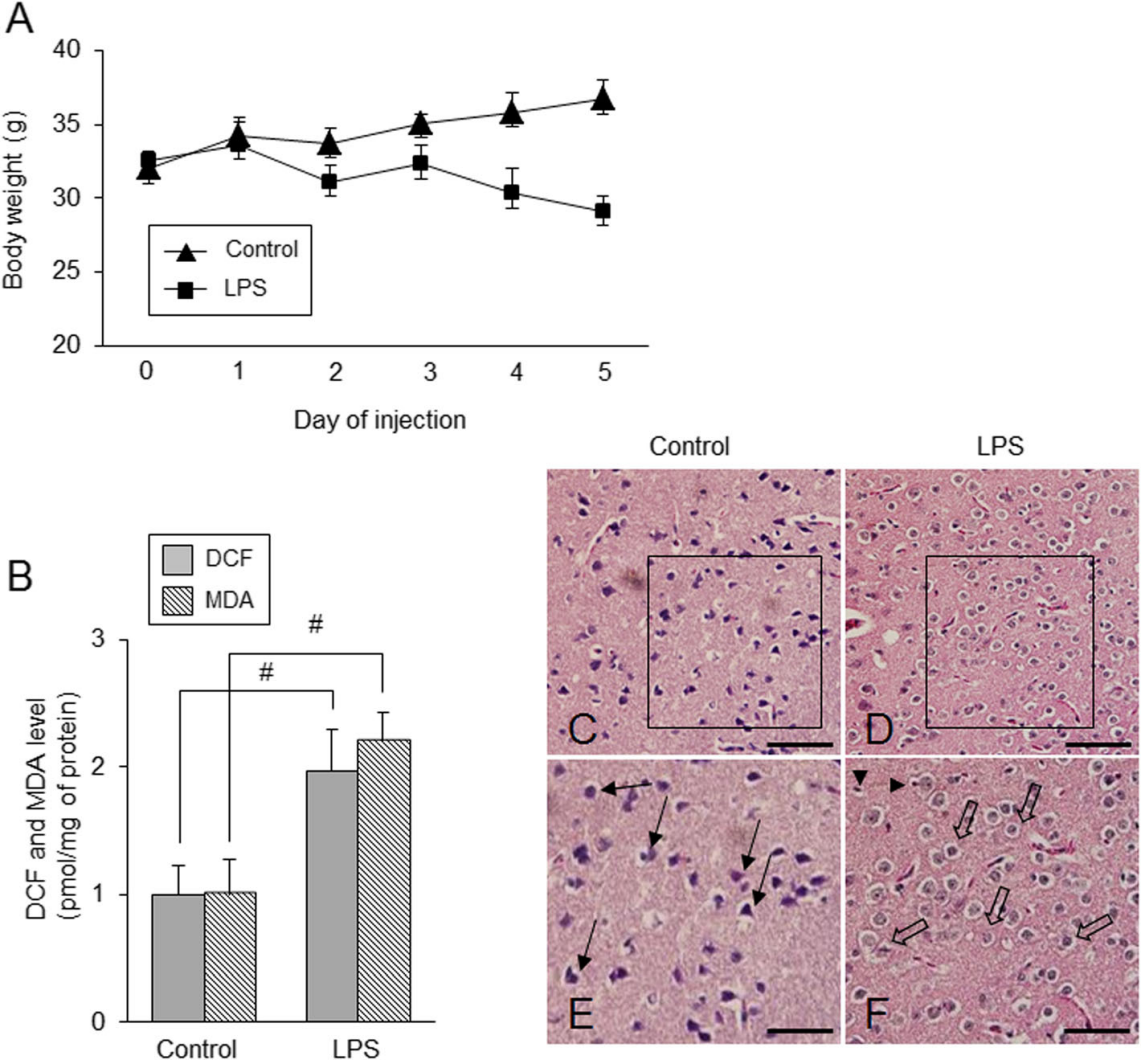
Body weight (**a**), reactive oxygen species (ROS) and lipid peroxidation (LPO) analyses (**b**), and representative photomicrographs of hematoxylin and eosin staining (**c**-**f**) in the cerebral cortices of control and lipopolysaccharide (LPS)-treated animals. LPS induced a reduction of body weight (**a**) and increases of DCF and MDA values (**b**). LPS treatment induced severe histopathological changes including swelling and vacuolation of nerve cells (**d** and **f**). Arrows indicate normal pyramidal cells with well-characterized dendrites (**e**). Open arrows indicate abnormal nerve cells that were swollen and vacuolated (**f**). Arrowheads indicate shrunken neuroglial cells (**f**). Magnified photos indicate square region. Scale bar = 100 μm (**c** and **d**), 200 μm (**e** and **f**). Data (*n* = 5) are shown as mean ± S.E.M. ^#^ *p* < 0.05 vs. control animal

LPS treatment induced a significant decrease of body weight (Fig. 1a). Body weights were 29.1 ± 0.98 and 36.7 ± 1.24 in control and LPS-treated animals, respectively, at 24 h after last injection. ROS and LPO values were measured as DCF and MDA levels, respectively. They were significantly increased in LPS-treated animals compare with those of control animals. DCF and MDA levels of control animals were set to 1. DCF and MDA levels in LPS-treated animals were 1.97 ± 0.33 and 2.21 ± 0.22, respectively (Fig. 1b). Figure 1c showed the histopathological changes in cerebral cortices after LPS exposure. Neuronal and neuroglial cells with normal morphology were observed in control animals. Most of the neurons had a typical pyramid shape with well-characterized dendrites (Fig. 1c and e). However, we observed severe histological changes in LPS-treated animals. Most of the nerve cells were swollen, with loss of their processes, surrounded by pericellular halos, and vacuolated (Fig. 1d and f). Neuroglial cells were shrunken and deeply stained (Fig. 1f).

**Fig. 2:**
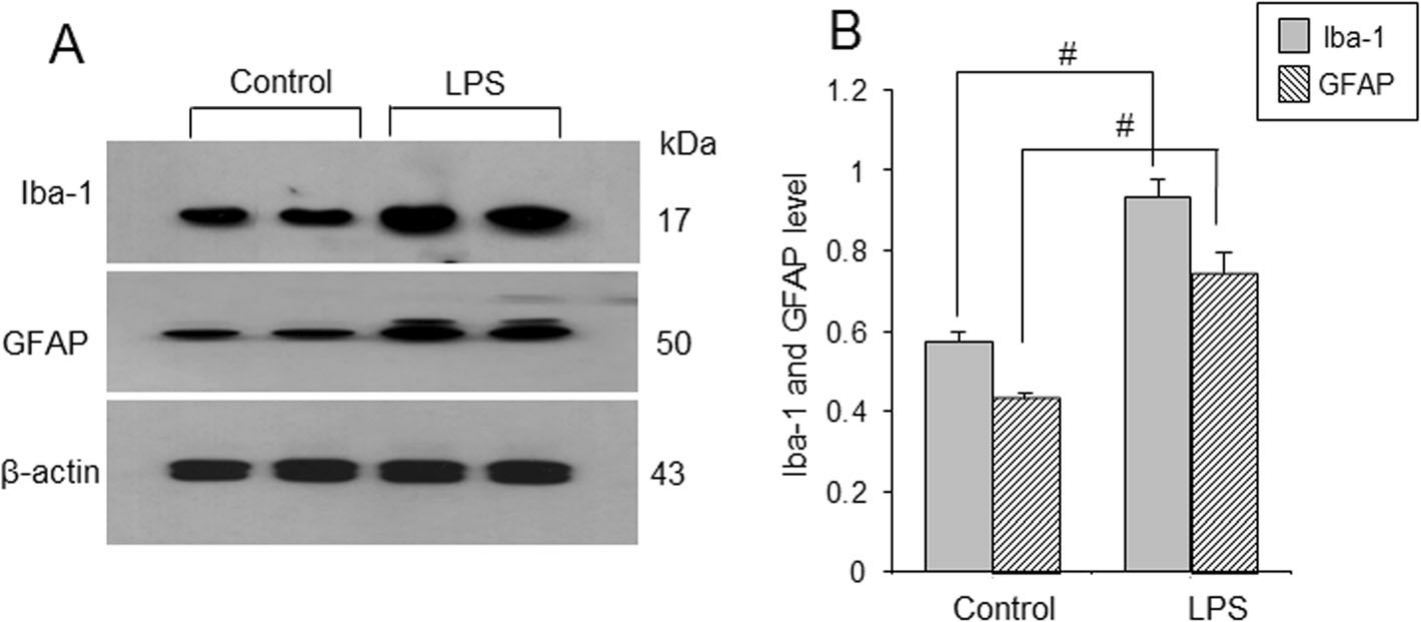
Western blot analysis of ionized calcium binding adaptor molecule-1 (Iba-1) and glial fibrillary acidic protein (GFAP) in cerebral cortices of control and lipopolysaccharide (LPS)-treated animals (**a**). Density values of Western blot analysis are expressed as a ratio of each protein intensity to β-actin intensity (**b**). Data (*n* = 5) are shown as mean ± S.E.M. ^#^ *p* < 0.05 vs. control animal

**Fig. 3:**
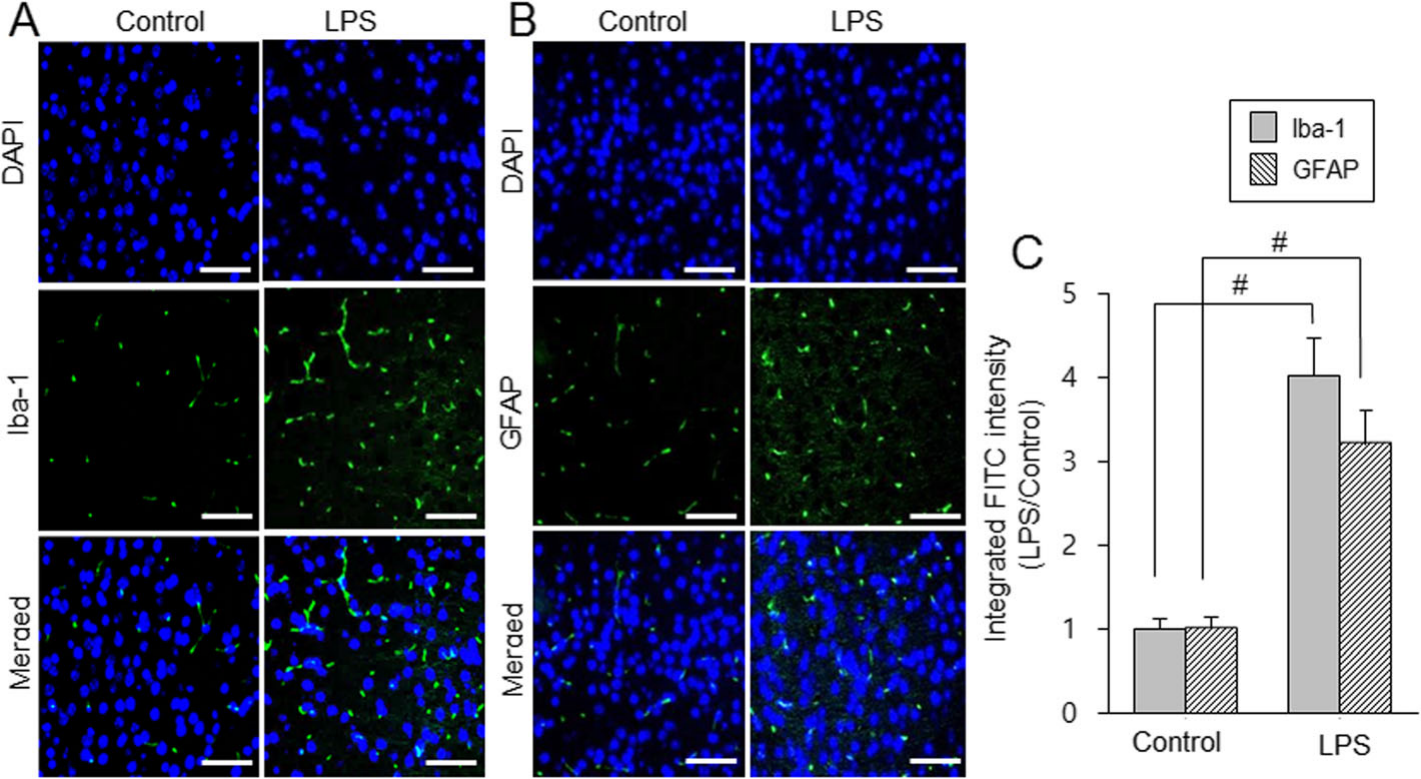
Double immunofluorescence labeling of ionized calcium-binding adaptor molecule-1 (Iba-1) and glial fibrillary acidic protein (GFAP) in cerebral cortices of control and lipopolysaccharide (LPS)-treated animals (**a** and **b**). Scale bar = 100 μm. Intensity values of fluorescence were expressed as ratio of intensity of LPS-treated to intensity of control animals (**c**). Intensity value of control animal was set to 1. Data (*n* = 5) are shown as mean ± S.E.M. ^#^ *p* < 0.05 vs. control animal

Iba-1 and GFAP expressions were investigated to elucidate activation of microglia and astrocytes. Western blot analysis showed that Iba-1 and GFAP expression levels were increased in LPS-treated animals (Fig. 2a). Iba-1 expression levels were 0.57 ± 0.02 in control animals and 0.93 ± 0.04 in LPS-treated animals. GFAP levels were 0.43 ± 0.01 and 0.75 ± 0.04 in control and LPS-treated animals, respectively (Fig. 2b). Moreover, immunofluorescence staining showed that Iba-1 and GFAP positive reactions increased in LPS-treated animals (Fig. 3a and b). Iba-1 and GFAP expression values were measured as a ratio of the intensity of control animals. Iba-1 and GFAP expression values in LPS-treated animals were 4.02 ± 0.44 and 3.21 ± 0.39, respectively (Fig. 3c).

**Fig. 4:**
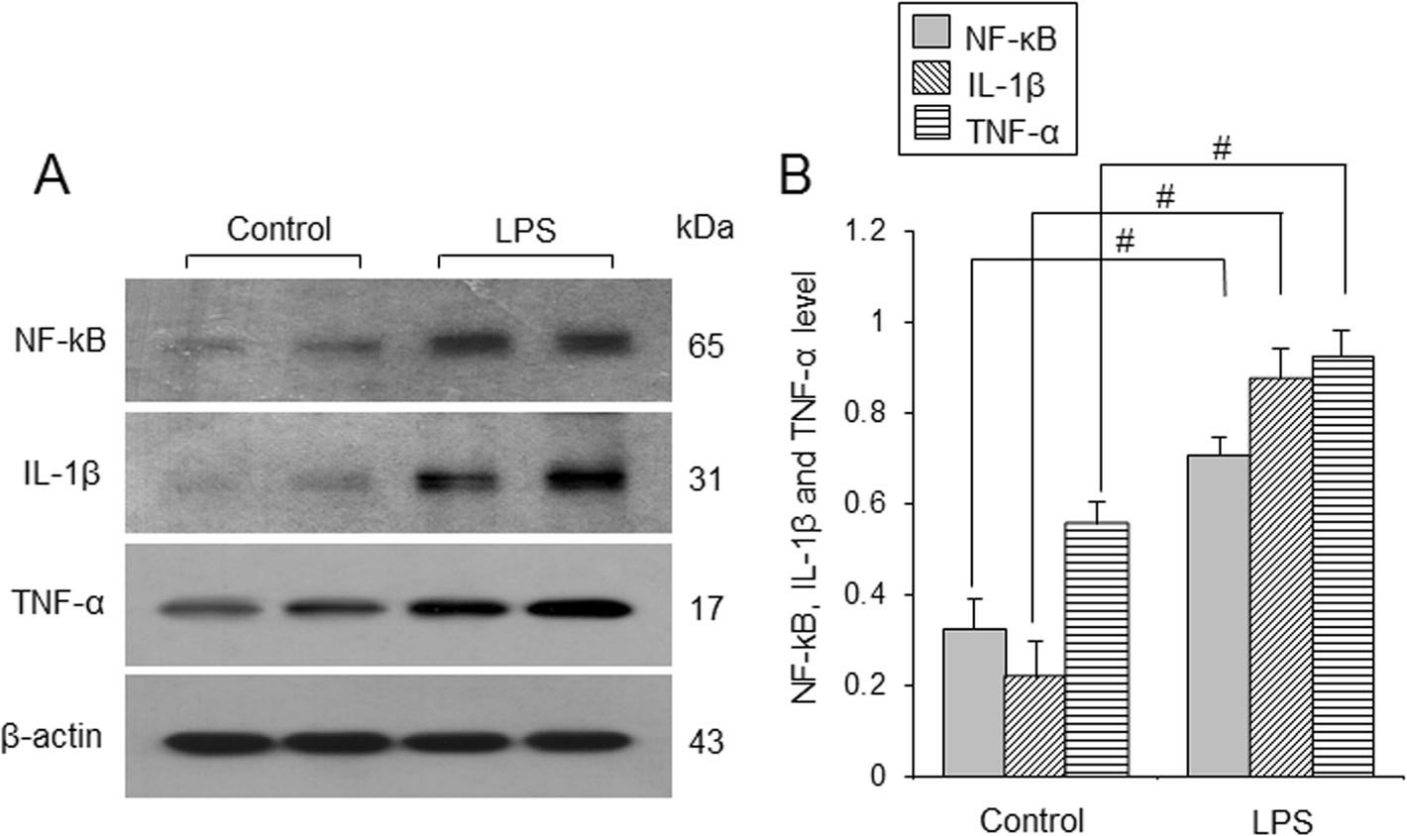
Western blot analysis of nuclear factor kappa B (NF-κB), interleukin-1β (IL-1β), and tumor necrosis factor-α (TNF-α) in the cerebral cortex of control and lipopolysaccharide (LPS)-treated animals (**a**). Density values of Western blot analysis are expressed as a ratio of each protein intensity to β-actin intensity (**b**). Data (*n* = 5) are shown as mean ± S.E.M. ^#^ *p* < 0.05 vs. control animal

**Fig. 5:**
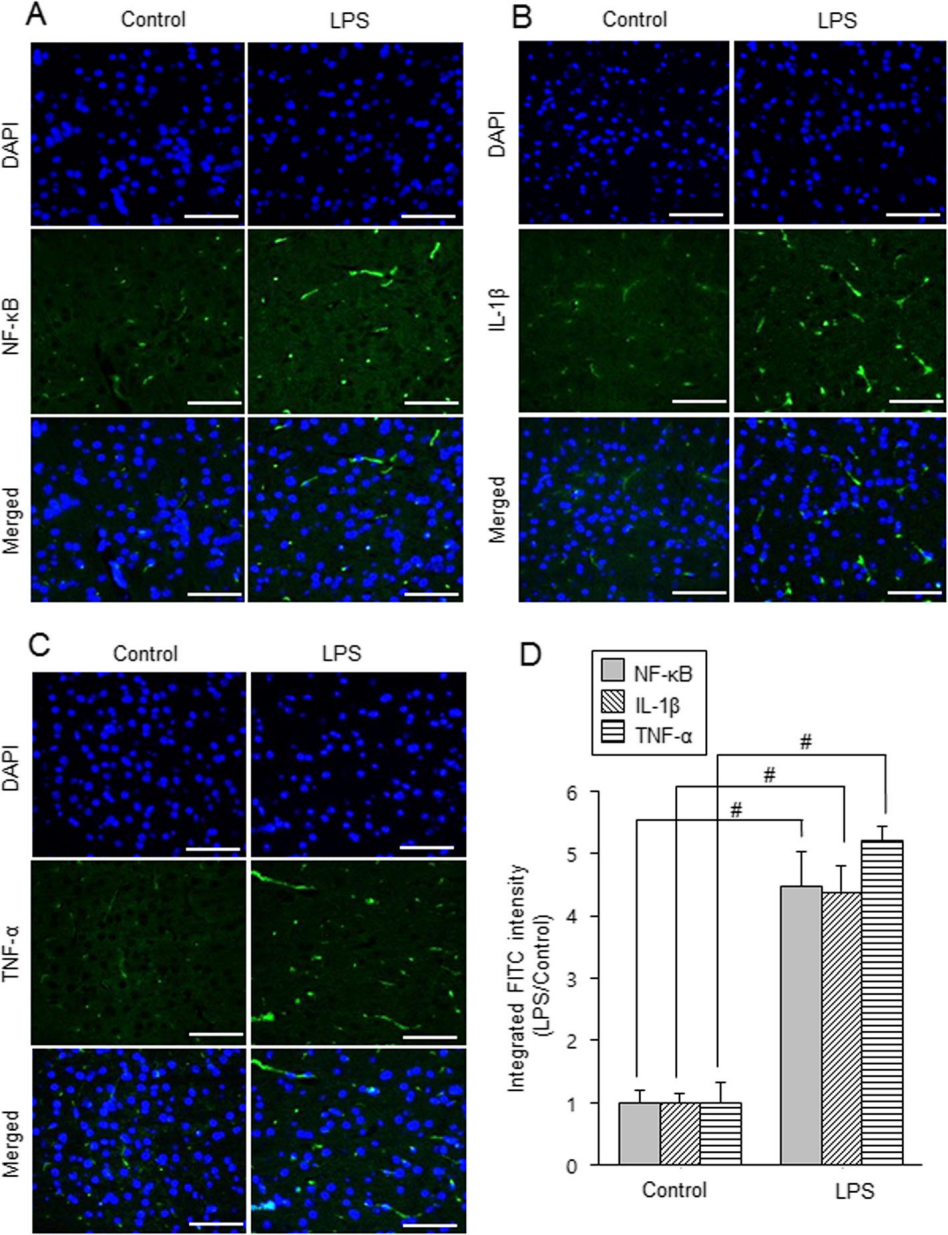
Double immunofluorescence labeling of nuclear factor kappa B (NF-κB), interleukin-1β (IL-1β), and tumor necrosis factor-α (TNF-α) in the cerebral cortex of control and lipopolysaccharide (LPS)-treated animals (**a**-**c**). Scale bar = 100 μm. Intensity values of fluorescence were expressed as ratio of intensity of LPS-treated animals to intensity of control animals (**d**). Intensity value of control animals was set to 1. Data (*n* = 5) are shown as mean ± S.E.M. ^#^ *p* < 0.05 vs. control animal

Figure 4a shows increases of NF-κB, IL-1β, and TNF-α in the cerebral cortices of LPS-treated animals. NF-κB levels were 0.32 ± 0.07 and 0.71 ± 0.04 in control animals and LPS-treated animals, respectively. IL-1β levels were 0.22 ± 0.08 in control animals and 0.87 ± 0.07 in LPS-treated animals. TNF-α levels were 0.55 ± 0.05 and 0.92 ± 0.06 in control animals and LPS-treated animals, respectively (Fig. 4b). Results of immunofluorescence staining confirmed increases of these proteins in LPS-treated animals (Fig. 5a-c). NF-κB was expressed in neuronal cell bodies and brain capillary cells and expression value was 4.47 ± 0.56 in LPS-treated animals. IL-1β and TNF-α was expressed in neuronal cell bodies expression values in LPS-treated animals were 4.40 ± 0.41 and 5.21 ± 0.23, respectively (Fig. 5d).

## Discussion

We clearly showed a reduction of body weight and histopathological changes such as, vacuolation, swollen cell body, and weakly stained cells in the cerebral cortex after LPS-induced neuroinflammation. Moreover, LPS induces over-production of oxidative stress and activation of neuroglia and leads to inflammatory response via NF-κB mediated inflammatory factors including IL-1β and TNF-α in cerebral cortex. We focused on activations of neuroglial cells and inflammatory mediators after LPS exposure in the cerebral cortex.

LPS activates astrocytes and microglia [28, 29]. Activated microglia release pro-inflammatory mediators and nitric oxide synthase, and lead to neuronal cells damage in degenerative disease [30, 31]. Moreover, oxidative stress leads to the release of inflammatory cytokines and nitric oxide from monocytes and macrophages. In this study, we assessed LPS-induced oxidative injury by ROS and lipid peroxidation analyses. We showed increases of DCF and MDA levels in the cerebral cortex after LPS exposure. LPS induces a decrease in membrane potential and an impairment in mitochondrial redox activity, leading to neuronal dysfunction and degeneration [32, 33]. Moreover, it also induces amyloid deposits and cognitive impairments [34, 35]. Our results showed that LPS activates microglia and astrocytes in the cerebral cortex and induces neuroinflammation. We observed increases of Iba-1 and GFAP expression in an LPS-treated cerebral cortex. Iba-1 and GFAP are used as indicators of activated microglia and astrocyte. Ibal-1 is up-regulated in microglia during nerve injury [36]. This study clearly showed an increase of Ibal-1 expression in damaged cerebral cortex caused by LPS exposure. Up-regulation of Ibal-1 indicates activation of microglia. Moreover, GFAP expression increases in central nervous system injury, neurodegenerative disease, and aging [37–39]. Increase of GFAP expression is also observed in neuroinflammatory disease [40]. Our results are in accordance with data of previous studies. We showed up-regulation of GFAP in the cerebral cortex of LPS-treated animals. Increase of GFAP demonstrates activation of astrocytes and activated neuroglial cells leads to release of inflammatory cytokines. These processes induce inflammatory response and ultimately lead to cell damage.

LPS treatment causes the activation of inflammatory mediators and leads to inflammatory responses. NF-κB releases pro-inflammatory cytokines such as TNF-α and IL-1β. This study showed increases of NF-κB, TNF-α, and IL-1β in a damaged cerebral cortex caused by LPS exposure. Activated NF-κB up-regulates pro-inflammatory cytokines, IL-1β and TNF-α. These cytokines participate in the progression of liver and lung injuries [41]. They were up-regulated in injuries. Moreover, IL-1β augments TNF-α-mediated inflammatory response [42]. TNF-α is related to excitotoxic and neuroinflammatory processes that occur in neurodegenerative disease such as ischemia and traumatic brain injury [43, 44]. We showed that LPS induces up-regulation of IL-1β and TNF- α in the cerebral cortex, and increases of these pro-inflammatory factors cause inflammatory responses. Our results demonstrated that LPS increases oxidative stress and activates neuroglial cells including microglia and astrocytes in the cerebral cortex. Moreover, LPS up-regulates NF-κB-mediated inflammatory factors such as IL-1β and TNF-α in the cerebral cortex. Previous studies reported that LPS mediates to neuroinflammation and neurodegeneration in the cerebral cortex of adult mice via the Akt/GSK-3β survival pathway [45, 46]. The LPS-mediated mechanism is very complex. Neuroinflammatory responses caused by LPS occur through various cell-signaling pathways. This study elucidated that LPS mediates inflammatory responses via NF-κB activation in the cerebral cortex.

## Conclusions

Our findings suggest that LPS administration induces neurotoxicity and neuroinflammation through activating neuroglia and NF-κB-mediated inflammatory factors in the cerebral cortex of adult mice. Therefore, this finding might provide a basic information about neuroinflammation induced by systemic LPS administration.

## Data Availability

The data that support the findings of this study are available on request from the corresponding author on reasonable request.

## Abbreviations

BCA: Bicinchoninic acid
DAPI: 4′,6-diamidino-2-phenylindole
DCF: Dichlorofluorescein
DCFH-DA: 2′,7′-dichlorodihydrofluorescein diacetate
FITC: Fluorescein isothiocyanate
GFAP: Glial fibrillary acidic protein
Iba-1: Ionized calcium-binding adaptor molecule-1
IL-1β: Interleukin-1β
LPO: Lipid peroxidation
LPS: Lipopolysaccharide
MDA: Malondialdehyde
NF-κB: Nuclear factor kappa B
NRF: National research foundation of Korea
PBS: Phosphate buffer saline
ROS: Reactive oxygen species
TBST: Tris-buffered saline containing 0.1% Tween-20
TNF-α: Tumor necrosis factor-α
